# Forensic Medicine
*"Essentials of Forensic Medicine, Toxicology, and Hygiene." G. Armand Semple, M.B., M.R.C.P. (London: Mr. Henry Renshaw.)


**Published:** 1890-09-06

**Authors:** 


### THE PRACTITIONER'S BOOKSHELF.

[Books for Review should be sent to The Editor, The Lodge, Porchester Square, W.]

### FORENSIC MEDICINE.*

This is one of Renshaw's "Illustrated Essentials" series, and is intended as a refresher before going in for the final examination. Taken as such it is a useful compendium of the chief points of medico-legal interest. The crush and worry of many subjects compressed into too short a time have given rise to endless series of cram books for examination. These are necessary evils, and the best we can do is to point out those which are of most service. This book is handy, well-printed, and contains a number of very fair illustrations. In the chapter on pregnancy there is a series of plates purporting to represent the absorption of the cervix during the enlargement of the uterus. These are illusory, because it is quite common for the cervix to remain totally unaltered in size to within a day or two of labour. We have repeatedly found this by actual investigation when resident in a maternity hospital. Among the certain signs of pregnancy is noted the uterine souffle. This may be heard in certain cases of uterine tumours, and is consequently not a

* "Essentials of Forensic Medicine, Toxicology, and Hygiene." G. Armand Semple, M.B., M.R.C.P. (London: Mr. Henry Renshaw.)

certain sign. There is no mention of Braxton Hicks' sign, viz., intermittent uterine contractions during pregnancy. On page 28 there is depicted a lateral view section of the female pelvic organs, the hollow viscera are represented as actual canals with the walls far apart. Except when mechanically distended this does not obtain in nature. All the tubes or potential cavity containing organs have their walls in contact unless the secretion or excretion peculiar to them is present.

In the account of the proceedings necessary to place a lunatic under restraint, we think it would be an improvement to print in full a form, such as is commonly made use of, properly filled up as a model; it would illustrate the required details better than a mere description or enumeration of the steps to be taken.

In the section on detection of blood stains, the Guaiacum test is described and cited as being extremely delicate. So it is, but it gives exactly the same reaction with a slice of fresh potato or scrapings of nearly any fresh vegetable suspended in water. It is useful only as strengthening the list of proofs.

The section on toxicology is fairly complete. Speaking of testing for arsenic, no mention is made of distilling the acidified products (with H Cl.), and then testing the distillate for the arsenic. This does extremely well where there is a considerable quantity present, and the tests are easier made because a clear fluid is used. In mercury poisoning it should be noted how small a dose is capable of producing intense effects on those suffering from Bright's disease. In the case of chronic lead poisoning abortion is very frequently induced. This is one of the methods by which abortion is sometimes procured.

In the section on opium poisoning the following occurs: "As regards the employment of belladonna preparations as antidotal to opium, it has been stated that these are worse than useless, as the presence of both poisons increases the effect of either." The antagonistic effects of opium and belladonna were worked out by Fothergill. In several cases of severe opium poisoning he effected the recovery of the patient by injecting sulphate of atropia subcutaneously; in one case as much as a grain was used. This succeeded when all other treatment of the usual kind had failed. The chapters on hygiene present a useful summary of the main facts of the science. In conclusion, we think Dr. Semple has written a compendium which compares very favourably with others on the same subjects. The style is easy, the compiler goes straight to the point, and by means of leaded type and breaking up into numerous paragraphs, the headings of the important sections are brought well before the eye. We can say of the work that it acts admirably as an indicating suggester to the subjects in larger works. There is a good index.

